# Evaluating a 30-Hour Training Program for Community Health Workers on 4Ms Implementation in FQHCs Using the Kirkpatrick Model

**DOI:** 10.3390/healthcare13212677

**Published:** 2025-10-23

**Authors:** Sweta Tewary, Cherell Cottrell-Daniels, Kevin Espinoza, Katherine Chung-Bridges, Diego I. Shmuels, Deborah Gracia, Joycelyn J Lawrence

**Affiliations:** 1 Jessie Trice Community Health Systems, Miami, FL 33142, USA; 2 Research Department, Health Choice Network, Miami, FL 33172, USA; 3 Borinquen Medical Centers Center, Inc., Miami, FL 33137, USA

**Keywords:** community health workers, aged, program evaluation, Electronic Health Records, age-friendly health systems and 4Ms care

## Abstract

Objective: To evaluate a 30 h educational program delivered to community health care workers (CHWs) involved in geriatric care within a primary care clinic, measure increase in knowledge, likelihood of using the education, and baseline results of geriatric screenings for patients 65 and older conducted by CHWs in their clinics. Methods: Design, Setting and Participants: This is an evaluation with a two-center, pre–post design study of a 30 h in-person educational program. The program used the Kirkpatrick model to evaluate the educational program. The study used quantitative and qualitative data collection with surveys measuring knowledge, feedback, content, and demographics of the participants and chart reviews to measure clinical implementation of 4Ms discussion. Qualitative data collection included a focus group and open-ended questions in the survey. Thematic analysis from focus groups explored the feedback from the educational program. Results: Twelve community health care workers (average age 40, 90% female) from two federally qualified health centers (FQHC) participated in the 30 h training program. Perceived knowledge improved after the completion of the training. Final exam scores after the training were also significant, indicating an improvement in content retention. Overall, 98% of participants described the training as “Excellent” and 96% rated excellent for the speakers who provided the training. Additionally, 83% suggested they would apply the training in their practice. Approximately 40% of chart reviews indicated the completion of the 4Ms (What Matters, Mentation, Medication, and Mobility) discussion with patients. Thematic analysis yielded two new practice dimensions: care provision and clinical documentation. The training resulted in organizational adaptation with the development of an intake form in the Electronic Health Record (EHR) to document the 4Ms. Conclusion: Results indicate improvement in all dimensions of the training with an emphasis on level 4, indicating wider organization adaptation of 4Ms discussion.

## 1. Introduction

With the growth in the aging population in the United States, there is an increased need for health care professionals to manage complexity in health problems, improve quality of life, and reduce the burden of care on the health care system. The risk of chronic conditions, such as dementia, heart disease, diabetes, and arthritis, also increases with age and can impact quality of life [1]. It is estimated that by 2034, the older adult population will be 77 million [2]. The changing demographics and health status indicators demonstrate the need for an increase in geriatric care. The American Geriatric Society advocates for an increased need for geriatric workforce and geriatric training [3]. The complexity and challenges of older adults and their health care needs will require the health care systems to adapt and ensure that health care professionals are equipped with the knowledge and clinical skills to meet the unique needs of older adults, including managing multimorbidity and polypharmacy, identifying cognitive and functional decline, and aligning care with patients’ goals and social contexts, all of which require specialized, age-friendly approaches [3,4]. Meeting this need could involve providing geriatric education through interprofessional collaboration and implementing programs to incentivize careers in geriatrics to provide holistic care to seniors. The Institute of Medicine has called for a comprehensive effort to build a workforce prepared to deliver high-quality care to older adults, highlighting the gaps in current education and training systems [4].

A community health worker (CHW) is an entry-level credential who can serve as a liaison between health services and the community to facilitate access to multiple services, including health education and supporting community resources [5]. CHWs are considered part of the public health and social services workforce in the United States [6]. They function in various capacities, including care coordination, health coaching, social support, conducting health assessments, and linking community members to services tackling social determinants of health [7]. Their roles also expand to providing culturally competent and relevant education, case management, immunization uptake, non-communicable disease screening, and providing outreach through home visits [8].

Research has shown that CHW-led interventions can improve health outcomes across diverse settings, particularly in low-resource communities [8,9,10]. A systematic review highlighted CHWs’ effectiveness in improving cancer screening rates, chronic disease management, post-hospital follow-up, and enhancing childhood immunization coverage [11]. Across the United States, several CHW models are being employed to address various public health and social services. For example, the Health Aide model in Alaska incorporates CHWs certified in behavioral, community, and dental health, offering care for Native communities with oversight from licensed clinicians [12]. Arizona’s Health Start program focuses on pre- and post-natal services for young families, utilizing CHWs for education and advocacy [13].

Employment opportunities and remuneration for CHWs vary based on education, skills, and experience [6]. Many community-based organizations, hospitals, and health systems hire CHWs for addressing social determinants of health, supporting preventative health programs, and patient education [14]. Certification of CHWs varies by state but typically involves a combination of a high school diploma or an associate degree, work or volunteer experience, and potentially an exam, which differs by state as well. Even though CHWs have a unique role in addressing health disparities, remuneration and sustainability have often remained a challenge [15]. A lack of standardized education results in inconsistent care quality and undermines their potential to address the unique needs of aging populations.

CHW programs have shown a positive return on investment. For example, programs such as the Individualized Management for Patient-Centered Targets, a community health worker intervention program addressing unmet social needs among individuals who are disadvantaged, have been shown to yield an annual return of USD 2.47 for every dollar invested [16]. However, they often lack formal and hands-on education in geriatric care and have limited access to professional development or career advancement in this area [17,18].

The age-friendly health system (AFHS) is an evidence-based practice framework using the 4Ms approach [19]. While this model has been implemented in clinical settings with positive results [20,21], little research has examined its integration into CHW training programs. Many CHWs are lay people with no formal training, and others learn on-the-job; there is no standardized, basic educational framework on which to draw from to train CHWs in geriatric care. Given the central role of CHWs in primary care, equipping them with age-friendly competencies is a promising strategy for expanding the reach and equity of geriatric care.

**Figure 1:**
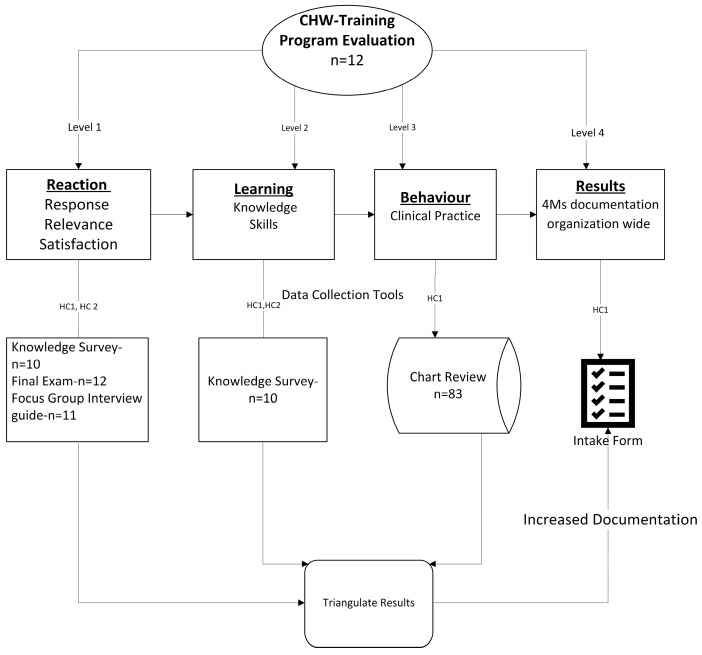
Training and evaluation process.

The program uses Kirkpatrick’s model to assess the effectiveness of training programs at four levels: (1) reaction of the program participants to the training experience, (2) program participants’ learning outcomes and increase in knowledge, (3) behavior change associated with the program participants’ change in clinical practice (whether the learning is transferred into practice in the workplace), and (4) results (the ultimate impact of training) [22,23,24,25] (see Figure 1). This approach provides a comprehensive structure for measuring how CHW training translates into improved clinical practice and outcomes for older adults.

This program was undertaken as a quality improvement (QI) initiative aimed at enhancing existing clinical processes and older adult patient care within two federally qualified centers (FQHCs), Health Center 1 (HC1) and Health Center 2 (HC2). Both health centers serve almost all northern communities of Miami Dade County, including the predominantly Hispanic communities, the Haitian American enclaves in Little Haiti and North Miami, and the African American communities of Overtown, Liberty City, and Opa-Locka. Total patient population for HC1 was approximately 30,000 with 4% aged 65 and older; it was predominantly male (61%) and 31% female. The majority of the patients (67%) identify as Black, followed by 23% Hispanic. At HC2, 42,079 individuals were served in 2024, with 67% being Hispanic and 28% Black; of these, 4.5% of adults were aged 65 and older, with a higher proportion of females (61%) compared to males (39%). Patients at both the health centers are typically employed in management, office administration, sales, transportation, construction, and health care practitioner roles. These are followed by education, food preparation, and building maintenance.

Patients mostly reside in zip code areas known for the highest prevalence of emergency department visits due to asthma-related issues associated with poor housing conditions, poor air quality, allergens, and maintenance in school building, highlighting the impact of environmental factors on health outcomes.

### Study Objectives

1. To evaluate the effectiveness of 30 h CHW training by measuring the increase in knowledge, likelihood of using the education, and trainee satisfaction.
2. To measure the documentation of 4Ms assessment by CHWs who are directly involved in geriatric care in primary care clinics.

## 2. Material and Methods

### 2.1. Study Design and Participants

We used a pre–post design to evaluate a 30 h CHW training provided at two FQHCs. The didactic training incorporated a two-step process: (1) Introductory 30 h CHW training followed by two separate meetings to help prepare for a state certification exam, and (2) follow-up guidance by a clinical team to implement 4Ms care for older adults 65 and above. Site selection and participants at the two health centers were based on purposive sampling. The inclusion criterion to participate in the program required full-time employment as a CHW in the health center. Therefore, the sample consisted of all CHWs enrolled in the program. CHWs included in the program did not have any prior experience or training with geriatric assessments with most of them having a high school degree. Data collection included primary and secondary data sources integrating both quantitative and qualitative approaches. Data were analyzed using SPSS 29 through Matched sample *T* test, chart review of EHR data, and thematic analysis of responses from focus groups.

### 2.2. Detailed Methods: Didactic Curriculum, Clinical, and Procedures

The introductory didactic curriculum content was provided by the Miami Dade Area Health Education Center (AHEC) in collaboration with the Florida Community Health Worker Coalition, a statewide multisector partnership dedicated to the promotion of the community health worker (CHW) profession and serving communities impacted by disparities through collaboration, educating the public, training, and leadership to improve health outcomes in Florida. Although CHWs are not formally classified as health care professionals, the COVID-19 pandemic led HRSA to federally recognize them as frontline workers, highlighting their vital contributions to community outreach, health education, and care coordination. The training plan adheres to two (2) of Florida’s legal and regulatory requirements. Florida acknowledges CHWs through Florida House Bill 183 (2021), titled Office of Minority Health and Health Equity, which was enacted into law and took effect on 1 July 2021. Also, Florida recognizes the voluntary certification process of the Florida Certification Board for Certified Community Health Worker. The Florida CHW certification is a state-recognized credential that validates a community health worker’s training and competency in providing outreach, education, and support to improve community health outcomes. This comprehensive introductory 30 h, five-day classroom training was delivered face to face through didactic exposure, case studies, group discussions, assignments, and an emphasis on teamwork to prepare CHWs to fully participate in their employers’ care teams and 4Ms care. After completion of training, CHWs have access to clinical data in accordance with federal HIPAA Privacy and Security Rules. Any access to protected health information (PHI) by CHWs is strictly limited to program requirements and occurs under HIPAA’s permitted uses and disclosures, including the public health exceptions, in addition to Florida’s Information Protection Act (FIPA, § 501.171, F.S.) and § 456.057 (confidentiality of medical records). These safeguards ensure that patient data are protected while CHWs are equipped to fulfill their frontline role in supporting care coordination, prevention, and community-based health interventions.

#### 2.2.1. Didactic Curriculum

The 30-Hour Geriatric Curriculum for CHWs in Primary Care Settings emphasizes the 11 core competencies across five modules, equipping CHWs to support older adults holistically. Module 1 introduces CHWs to the basics of the role, interpersonal and communication skills, understanding social determinants of health (SDoH), and care coordination, covering community engagement, assessment skills, and communication. Module 2 focuses on elder health foundations, including the aging process, chronic disease, mental health, and the 4Ms—supporting competencies in health education, behavioral health awareness, and chronic disease support. Module 3 teaches CHWs to locate and navigate resources, overcome access barriers, and coordinate services, reinforcing care coordination, advocacy, and data collection/reporting. Module 4 covers professional responsibilities, ethics, documentation, and self-care, aligning with ethics, professionalism, and advocacy. Finally, Module 5 focuses on advocacy and empowerment, teaching CHWs to amplify patient voices, promote health equity, and facilitate access to resources, which strengthens advocacy, community engagement, and communication. Together, these modules ensure CHWs are prepared to apply all 11 competencies—community engagement, health education, care coordination, communication skills, advocacy, assessment skills, data collection and reporting, behavioral health awareness, medication literacy, chronic disease support, and ethics/professionalism—to improve the health and well-being of older adults in both clinical and community settings.

The training was facilitated by a group of educators with a strong background in health education and elder care and extensive experience training individuals to become CHWs. The training team was led by an experienced leader of 17 years of work in community health, social services, and CHW development. Educational background of the team leader included a dual master’s degree in social work and public health, as well as a Ph.D. in Public Health.

#### 2.2.2. Clinical and Procedures

The 4Ms care in the program weas measured through a 4Ms intake form (Supplementary Materials S1—4Ms Intake Form), screening tools, and visit summary in the EHR. Questions on what matters, part of the 4Ms intake form, were asked by the CHWs and documented in the notes section through smart phrases within the Care coordinator/Compass Rose in the EHR. Medication reviews were completed by the medical assistants at each visit and reviewed by the physicians and medical residents. This information was documented in the rooming section and visible to clinical providers through multiple screens such as the assessment, plan of care, and wrap-up section in Compass Ross within the EHR. CHW also used the 4Ms intake form to ask additional questions related to medication management. The responses were documented in the notes section through smart phrases in Care coordinator/Compass Rose within the EHR.

Mentation was measured through dementia and depression screenings. We used Minicog for dementia screening, which required a clock drawing and a three-minute assessment for cognitive impairment, either by a physician or a CHW. Responses were scored on correct word recall and the accuracy of the clock drawing. Patients with a score of ≤2 on a 5-point scale required a referral to a neurologist for further evaluation. The screening tool was accessed and documented through the screening tab within the EHR. Dementia questions included in the 4Ms intake form were also asked by CHWs and documented in the notes section through smart phrases in Care coordinator/Compass Rose within the EHR.

Depression was measured through the Patient Health Questionnaire (PHQ-9), a screening instrument used for screening and diagnosing depression, completed mostly by the medical assistants. The PHQ-9 was accessed and documented through screenings within the EHR. Patients were typically administered the PHQ-9 at each visit to monitor the symptoms of depression with scores ranging from 0 to 27. Scores ranging between 10 and 27 were referred to the behavioral health service, psychiatric intervention, and crises intervention. In addition to PHQ-9 completion, CHWs completed depression questions through the 4Ms intake form and used smart phrases to document the information in the notes section of the Care coordinator/Compass Rose within the EHR.

Mobility required documentation of falls risk through multiple methods by the clinical team. The medical assistants discussed fall risk questions and documented them in the EHR. These questions were (1) one or more falls in the last year (2) how many times and (3) was the patient injured in any fall? (4) feels unsteady when walking, and (5) worried about falling? The CHWs and physicians also completed Timed Up and Go (TUG) assessments, a clinical tool to assess mobility, balance, and fall risk. TUG was accessed through the screening template within EHR. Several other fall-related questions were asked by the CHWs using the 4Ms intake form. The responses were documented with information on other “Ms” in the Care coordinator/Compass Rose within the EHR.

Follow-up guidance on implementing 4Ms care was provided by an interprofessional primary care team including physicians, a diabetes educator, and a faculty researcher. CHWs were integrated into the primary care teams to enhance patient-centered care by supporting screenings for 4Ms and social determinants of health (SDoH), follow-up between visits, and referrals for community-based resources that address SDoH. Patients 65 years and older were assigned to the CHWs to discuss and guide conversations around 4Ms care and discuss what matters most, medication, mentation, and mobility. The CHWs shared 4Ms documentation through weekly virtual meetings with the clinical team. Challenges in EHR documentation, timeliness of completion, and urgent referrals were discussed in the meetings. In addition, CHWs discussed positive screens on dementia, depression, mobility, and what matters conversation to plan follow-ups with the patients through a care plan, phone calls, notes, and care management documentation in the EHR. This approach helped with the formation of the patient’s care plan and also strengthened continuity of care and helped improve patient outcomes. See Supplementary Materials S1—4Ms Intake Form.

### 2.3. Data Collection and Evaluation

Our program evaluation used all 4 levels of Kirkpatrick’s model to measure the reaction, learning outcome, behavior change with respect to performance delivery, and results indicating impact of training. See Figure 1. The first two levels, Levels 1 and 2, are applicable to both health centers HC1 and HC2. Levels 3 and 4 are only applicable to the participating center HC1. We used mixed methods to analyze focus groups, EHR data, and self-administered surveys. Data from focus groups were analyzed through thematic analysis using a focus group interview guide for Levels 1 and 2 of Kirkpatrick’s model. The semi-structured focus group interview guide was developed to explore CHWs’ experiences with the training, their current roles, and the support needed to integrate training content into practice. Key domains included the following: (1) professional background and responsibilities; (2) perceptions and application of the AHEC CHW training; (3) barriers and facilitators to implementation; and (4) recommendations for improving training and organizational support. The guide included open-ended questions to encourage discussion on how CHWs apply training in real-world settings, challenges encountered, and opportunities for ongoing professional development. See Supplementary Materials S3.3. Eleven of the twelve CHWs participated in the focus groups. Saturation was not sought after since this was a quality improvement initiative and hearing the perspective from all of the CHWs that participated in the training was relevant to understand the training experience, applicability to clinical practice, and future training needs.

### 2.4. Data Collection Instruments

(a) Program Evaluation Survey: This questionnaire included questions about the feedback related to the content of the training and the speakers delivering the educational information. Supplementary Materials S3.1.
(b) Knowledge Survey: The questionnaire included items on demographics, applicability of the content, likelihood of application, and knowledge gain. Supplementary Materials S3.2.
(c) Final Exam: A content-based exam was also administered after completion of the 30 h training. Data were analyzed using frequency and percentages. Supplementary Materials S2.
(d) Focus Groups Interview Guide: Qualitative data were analyzed through thematic analysis to identify common themes and insights regarding the training’s impact on clinical practice. Supplementary Materials S3.3.
(e) Chart Reviews: To evaluate the clinical implementation of the 4Ms care, secondary data were extracted from the EHR to observe 4Ms completion.

Level 1 Reaction (HC1 and HC2)—The first level of the Kirkpatrick program evaluation is related to the response of program participants to the training experience. The response of the participants was evaluated through two different surveys. Supplementary Materials S3.1—Program Evaluation Survey covered 12 closed-ended questions using a 5-point Likert scale (1 = Poor, 2 = Fair, 3 = Good, 4 = Very Good, 5 = Excellent) for nine questions and a 3-point Likert scale (1 = Not at all, 2= Somewhat Did, 3 = Definitely Did) for two questions. Two open-ended questions were included to measure new knowledge gained and suggestions. Supplementary Materials S3.2—Knowledge Survey, Question 18 assessed participants’ likelihood of applying the information, and suggestions to improve the training. These questions addressed (1) the likelihood of applicability of knowledge, Likert scale: very likely, somewhat likely, and not likely; and (2) suggestions to improve the training, open-ended responses. Qualitative data through focus groups also explored feedback on applicability of the training (Supplementary Materials S3.3 Focus Group-Interview Guide, Questions 6 and 7).

Level 2 Learning (HC1 and HC2)—In the second level of Kirkpatrick’s model, the learning outcome was evaluated through two questions in Supplementary Materials S3.2 (Questions 16 and 17)—Knowledge survey: Now that you have completed the training, how much knowledge do you feel you have in this training topic? Likert scale: Low, Medium, High; final exam score, and open-ended question related to skills about the training from Supplementary Materials S3.3 Focus Group-Interview Guide, Questions 9 and 11.

Level 3 (HC1) Behavior accounts for measures of change in the skills or performance of participants after completing the training. We assessed if the learning was transformed into clinical practice through 4Ms documentation in the patients’ charts. This discussion was initiated through an intake form with a checklist incorporating multiple questions in HC1. The CHWs used this checklist while providing care to older adults and documenting the responses in the EHR as well as in Excel for clinical discussion, see Supplementary Materials S1. In the 4th level of evaluation (HC1), we assessed the results of the training by improving the quality of care for older adults at additional sites, incorporating an intake form in the her, as well as preparing the CHWs for the state-level exam.

### 2.5. Program Fidelity (HC1)

Program fidelity was assessed through weekly assessments, observation of CHWs in the health center, and review of patient documentation. The CHWs maintained documentation of their patients and discussed the workflow and documentation in weekly meetings. There were constant supervision and education by the providers and EHR navigators to help with clinical integration and the upload of information in the EHR.

### 2.6. Ethical Considerations

All participants were required to complete the final exam and data collection surveys. Data were de-identified to protect the confidentiality of the participants. Data from chart reviews and focus groups were also de-identified and aggregated support or analysis. The project was completed as part of an internal evaluation rather than human subjects research. In accordance with institutional and federal guidelines, activities conducted solely for evaluation—where the intent is to improve services and not to publish or generalize findings—do not require formal informed consent. However, participants were informed of the purpose of the evaluation; their participation was voluntary with clear communication that involvement or non-involvement would not affect employment or training status. All data were de-identified prior to analysis, and no individual-level identifiers were included in reports or presentations. Confidentiality was maintained by securely storing data on password-protected servers with access limited to the evaluation team. These measures ensured that participants’ privacy and autonomy were respected, even though this was conducted as a quality improvement initiative rather than human subjects research. Our program did not involve experimental interventions, randomization, or a systematic investigation designed to contribute to generalizable knowledge. As such, it does not meet the federal definition of research involving human subjects under 45 CFR 46.102.

## 3. Results

All twelve CHWs who registered for the training completed both the feedback assessment and final exam administered by the training organization, AHEC, resulting in a 100% response rate. However, only ten participants completed the knowledge survey, yielding a response rate of 83% See Figure 1. The average age of CHWs was 40 with 66% female. Table 1 provides the results of questions related to the likelihood of the use of education. Eighty three percent (83%) of participants responded that they would apply the training in their practice. Implementation of 4Ms was led by CHWs at three sites at HC1 with clinical supervision as needed.

### 3.1. Level 1 Participants Response, Relevance, and Satisfaction

Overall, the training course was positively evaluated (median 4 Excellent; IQR 1). Table 2 provides the distribution of ratings of questions regarding the content and speakers. Approximately 98% rated the training excellent. Feedback from qualitative questions informed that the CHWs received additional resources, knowledge, screening tools, teaching skills, knowledge of the importance of advocacy, social determinants of health, and assistance to help older adults. Participants also mentioned the ability to understand the importance of the CHW profession in health care settings. The feedback about the speakers was also positive, with 96% providing excellent ratings. Some other comments and suggestions were (1) educators were passionate and empathetic, (2) will continue to improve themselves and help the elderly population, and (3) will be motivated to be a great educator/advocate for their working institution.

The focus group discussion evaluated the relevance of the training for both health centers. The roles of CHWs varied across the FQHCs, with CHWs at HC1 focused more on community outreach and fieldwork, particularly with older adults, while CHWs at HC2 operated primarily within the clinic setting, handling tasks such as patient registration, eligibility screening, and service referrals. These differing operational contexts influenced how the CHWs applied their training. At HC1, CHWs emphasized using interpersonal strategies, such as active listening and open-ended questions, to uncover patient needs during community interactions. In contrast, CHWs at Health Center 2 applied training to improve internal processes, such as integrating SDoH screenings to identify and address patient needs proactively.

Feedback from the focus group reflected both appreciation and areas for improvement. CHWs from HC1 valued the sessions but expressed a desire for more in-person, hands-on practice with tools within the EHR to improve both usability and team collaboration. Meanwhile, HC2 CHWs recommended extending the training duration and called for clearer pathways to certification to enhance professional development. In terms of patient interaction, HC1 CHWs shared examples of building strong, personal connections with patients—often helping them navigate urgent resource needs, such as housing or transportation. CHWs at HC2 described more structured efforts, such as conducting outreach before appointments and coordinating across departments to streamline patient care. Despite similar challenges related to limited or outdated resource directories, CHWs in both settings remained committed to their roles. CHWs at HC1 highlighted the emotional fulfillment of helping community members directly, while CHWs at HC2 voiced frustration that their work was sometimes underrecognized within the clinical environment.

### 3.2. Level 2 Knowledge and Skills (HC1 and HC2)

In this step, we evaluated the knowledge pre–post training, See Table 3. The knowledge survey question evaluated CHWs’ knowledge through a 4-point Likert scale with None, Low, Moderate, and High responses. The knowledge survey was completed by 10 participants. Data distribution for the differences in pre- and post- scores was not normally distributed, Kolmogorov–Smirnov test, *p* < 0.05. Since the data were not normally distributed, we used the Wilcoxon signed-rank test to analyze the differences between the scores. The post-test ranks were significantly higher than the pre-test ranks; Z = −2.41, *p* < 0.05 (See Table 3) with an effect size of 0.91. (See Table 2). The final exam scores for 12 participants reviewed and scored through the AHEC team were analyzed through the Wilcoxon signed-rank test. The post-test scores were higher than the pre-test and demonstrated an increase in knowledge post-training with a Z = −2.949, *p* < 0.05 with an effect size of 0.89.

Eleven CHWs participated in focus groups across the two health centers. Focus group questions 9 and 11 discussed whether the training provided essential skills and learning relevant to their job. Discussion revealed that even though there were common themes across the focus groups, notable differences in learning emerged in areas such as role responsibilities, approaches to applying training, training needs, methods of patient interaction, resource limitations, and participants’ perceptions of their roles. These distinctions highlight the varied experiences and perspectives based on job functions and organizational contexts, underscoring the need for tailored strategies to address the unique challenges and opportunities within each group.

### 3.3. Level 3 Measures of Change (HC1)

Participants provided 4Ms care in one FQHC at three sites: The participants who received 4Ms care were older adults with a mean age of 72 years (min = 65, max = 74), See Table 3. All patients over the age of 65 were recruited through three sites of one FQHC. Implementation of 4Ms: Retrospective chart review of older adults from February to July 2025 indicates a 40% completion on all 4Ms; 100% completion on medication review, mobility, and matters most (See Table 4).

### 3.4. Level 4 Results (HC1)

**Figure 2:**
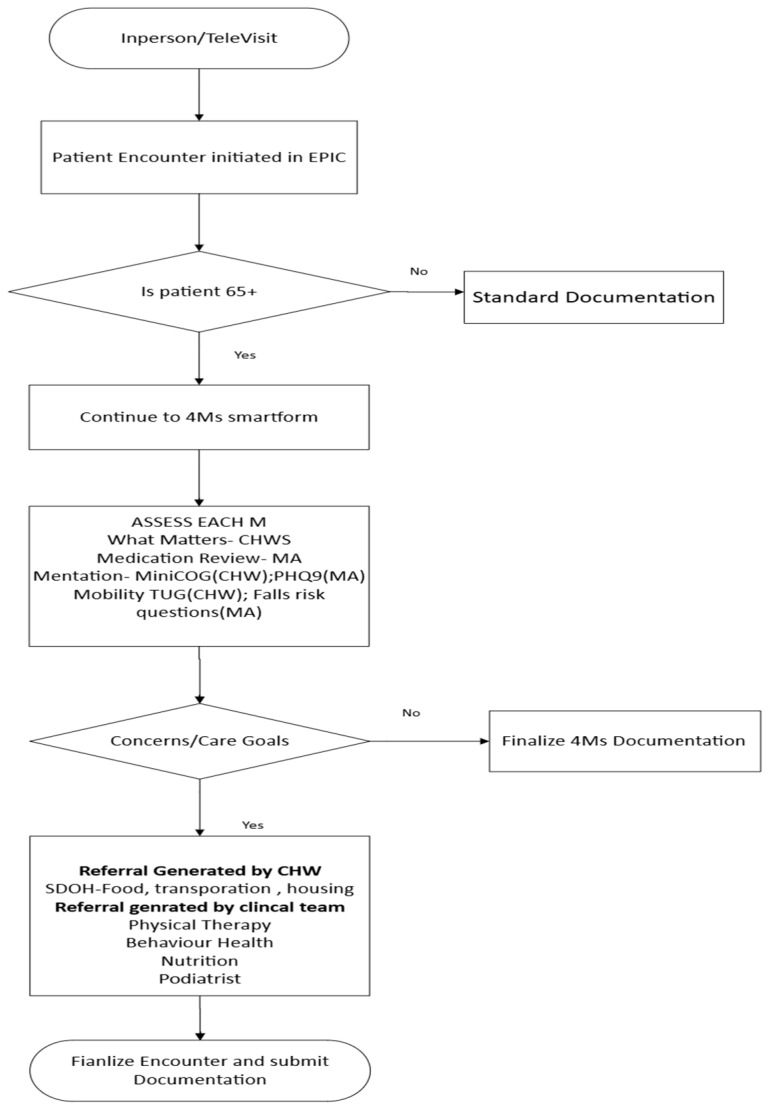
4Ms implementation organization-wide.

This level measures the effect of training on the organization in terms of improved quality in providing 4Ms care as well as preparedness for CHWs for the state-level exam. We were not able to calculate return on investment as we do not have longitudinal data; however, we were able to improve our workflow process by increasing awareness of AFHS through the CHW training, incorporating an intake form in the EHR to document the 4Ms discussion, and increased coding of the screening measures. Even though the form was administered by few CHWs, the work was highlighted for organization-wide adaptation (See Figure 2).

## 4. Discussion

This paper presents the evaluation of a 30 h in-person training designed to improve geriatric knowledge and pursue state certification. The program measures training evaluation and its impact in clinical settings while implementing AFHS and 4Ms. Our training was structured around the curriculum for state certification with modifications in content incorporating AFHS and 4MS care, critical components for delivering high-quality geriatric care [4,21].

The evaluation was guided by Kirkpatrick’s model [22,24]. Training feedback suggests a positive impact on all four dimensions. Our first dimension, Level 1 (Reaction), highlighted participants’ satisfaction with the training content as well as the speakers through surveys and focus groups. Participants expressed appreciation of the new content with request for booster sessions.

The second dimension, Level 2 (Learning) demonstrated post-training improvement in knowledge and application of 4Ms screening after the training. Results from the knowledge questionnaire indicate significant improvement in post-evaluation scores. Scores from the final exam also indicate an improvement in post-exam scores, suggesting retention of the training content. Qualitative thematic analysis through a focus group resulted in feedback of valuable insights and knowledge about the training contents.

While the training addressed CHW skills, it also provided a platform for participants to express their clinical challenges in patient care through our third dimension, Level 3 (Behavior), highlighting behavior change. CHWs conducted 4Ms screenings and discussed findings with the providers and care team. This helped with the formation of the patient’s care plan. There was 100% completion on specific 3Ms and 33% completion on all 4Ms for older adults 65 and above. Screenings such as TUG and MINI COG for falls risk and dementia, respectively, were not completed due to multiple factors, such as (1) the content was not covered in our first cohort training, (2) being an FQHC, patients are limited to a 15 min visit, making it difficult for the provider and the clinical team to complete a comprehensive 4Ms assessment unless needed, and (3) challenges in workflow integration due to difficulty in integrating 4Ms documenting form in the EHR. Additionally, data extraction related to 4Ms care remains a persistent challenge due to the lack of a structured EHR form specifically designed to capture and document 4Ms elements systematically.

At the results stage of our model (Level 4, Results), we supported CHWs for state certification by organizing follow-up training during the year. We included 4Ms screening in our clinical workflow which is facilitated by medical students and residents. Additionally, we incorporated and modified our 4Ms screening tool in the EHR system and applied for Committed to Care Excellence certification through the Institute of Health Care Improvement. We also incorporated a patient satisfaction question specific to 4Ms screening in our feedback survey. However, while these initiatives indicate improvement in advanced system-level changes, data extraction remains a challenge due to its unstructured format.

This program has several strengths. It addresses a pressing workforce gap by operationalizing the age-friendly health system (AFHS) 4Ms through the CHW training in federally qualified health centers, using an interprofessional approach and EHR-embedded tools. The evaluation used mixed methods (surveys, proctored knowledge assessment, focus groups, and EHR review), enabling triangulation of acceptability, learning, behavior change, and early practice outcomes. The program also produced replicable materials (curriculum, intake templates, documentation workflow) and actionable implementation insights for scale-up. Nonetheless, limitations include the pre–post, without a comparison group, purposive sampling, a modest sample of 12 participants from two FQHCs in one region that may limit generalizability, and a short follow-up that prevents assessment of durable effects or patient-level outcomes. Some measures relied on self-report and may be vulnerable to social desirability; EHR extraction constraints may have led to incomplete capture of 4Ms processes; and participation by motivated CHWs introduces potential selection bias.

Our findings align with previous CHW training studies demonstrating improvements in knowledge, confidence, and clinical contributions following structured educational interventions. For example, systematic reviews and randomized trials have documented that CHW-led programs can enhance chronic disease management, preventive screenings, and patient engagement across diverse community and clinical settings [6,10,11]. Like these studies, our program produced significant pre–post improvements in CHW knowledge and early evidence of behavior change, as reflected by the integration of 4Ms discussions into clinical encounters. However, unlike many prior interventions that focused primarily on disease-specific content (e.g., diabetes, maternal health), this initiative emphasized geriatric competencies and age-friendly care—an area in which CHW training remains limited.

With respect to 4Ms implementation, previous studies in primary care have shown that structured workflows, EHR templates, and team-based approaches are critical for successful adoption [20,21]. Our early implementation results—particularly the high completion rates for medication review, mobility, and what matters—mirror findings from these prior initiatives, which suggest that targeted training coupled with documentation tools can facilitate the uptake of age-friendly care practices. However, challenges around mentation screening and comprehensive 4Ms completion have also been reported as barriers in other settings, underscoring the need for intentional workflow redesign and infrastructure support.

To address these barriers, we propose three strategies: (1) embedding standardized 4Ms intake forms and “smart phrases” within the EHR to streamline documentation and facilitate data extraction; (2) allocating protected time for CHWs to complete assessments in advance of provider encounters, thereby easing time constraints during visits; and (3) establishing interdisciplinary case reviews where CHWs can share 4Ms findings with the care team to inform care planning. These strategies may help standardize 4Ms documentation as part of routine practice, improve communication across roles, and strengthen accountability for follow-up actions.

Overall, the findings demonstrate the feasibility and early effectiveness of implementing a CHW training program focused on geriatric care, aligned with national efforts to expand the geriatrics workforce and improve outcomes for older adults [3,5]. However, sustaining and scaling this model will require continued organizational support, structured documentation practices, and attention to the workflow constraints faced by FQHCs.

## 5. Conclusions

The lack of professional growth opportunities in geriatrics for CHWs is a significant barrier. Without clear career advancement pathways or specialized training, CHWs may not feel motivated to remain in the field or develop expertise in geriatric care. This program addressed this gap by incorporating a 30 h training for CHWs to advance their knowledge in geriatric care. Follow-up booster sessions in the training pathway included information on screening tools to help CHWs prepare for state certifications. State certification will allow CHWs to (1) advance their career and become more adept at recognizing and addressing the needs of older adults; (2) develop career pathways: establish professional growth opportunities in geriatrics, such as offering advanced certifications, leadership positions, or other incentives to stay in the field, and (3) expand educational and evaluation research on the effectiveness of CHW-led interventions specifically targeting older adults. Future studies should describe cost-effective methods to incorporate 4Ms assessment outside of annual wellness visits and methodologies to extract unstructured data.

## Figures and Tables

**Table 1:** Participants demographics (HC1 and HC2).

Characteristic (n = 12)	Subcategory	n (%)
Age (Average)	40	
Gender	Female	8 (67%)
	Male	1 (8%)
	Not Reported	3(25%)
Race	White	5 (42%)
	Black	4 (33%)
	Other	1 (8%)
	Not Reported	2 (17%)
Likelihood to Use the Education	Very Likely	10 (83%)
	Not Reported	2 (17%)
Knowledge Before Training	None	2 (17%)
	Low	1 (8%)
	Moderate	4 (33%)
	High	3 (25%)
	Not Reported	2 (17%)
Knowledge After Training	High	10 (83%)
	Not Reported	2 (17%)

**Table 2:** (HC1 and HC2) Descriptive statistics of ratings for training content and speakers.

Question	Excellent	Very Good	Good	Total (n)
The content presented was appropriate	10 (100%)	0 (0%)	0 (0%)	10
The information could be applied to own practice	10 (100%)	0 (0%)	0 (0%)	10
Helpful in achieving professional goals	10 (100%)	0 (0%)	0 (0%)	10
Speaker 1 had clear knowledge	9 (95%)	1 (5%)	0 (0%)	10
Speaker 1 content matched objectives	9 (95%)	1 (5%)	0 (0%)	10
Speaker 1 communicated effectively	10 (100%)	0 (0%)	0 (0%)	10
Speaker 2 had clear knowledge	10 (100%)	0 (0%)	0 (0%)	10
Speaker 2 content matched objectives	10 (100%)	0 (0%)	0 (0%)	10
Speaker 2 communicated effectively	10 (100%)	0 (0%)	0 (0%)	10

**Table 3:** Knowledge survey training pre–post results. (a) (Wilcoxon signed-rank test).

Comparison Outcome	Frequency	Mean Rank	Sum of Ranks	Z	*p*-Value
a. Positive Ranks	6	3.5	21	−2.23	0.026
b. Negative Ranks	0	—	0		
c. No Change (Ties)	4	—	—		

Notes: a. post-training scores > pre-training scores. b. post-training scores < pre-training scores. c. post-training scores = pre-training scores.

**Table 4:** 4Ms completion —retrospective chart review.

4M Category	# Completed	% Completed
Medication Review of Reconciliation	83	100
Mentation Depression	69	83
Mentation Dementia	33	40
Mobility Addressed (Falls Questions, Tug Test, Exercise, PT Referral)	83	100
Matters Most	83	100
All 4Ms Were Completed	33	40

Note. n = 83. “All 4Ms Were Completed” indicates patients for whom all elements of the 4Ms framework were addressed.

## Data Availability

The datasets presented in this article are not readily available because the data cannot be provided due to institutional policies as well as privacy and ethical concerns. Data may be available from the corresponding author upon reasonable request with appropriate data sharing agreements.

## Supplementary Materials

The following supporting information can be downloaded at https://www.mdpi.com/article/10.3390/healthcare13212677/s1.
