# Supplementary material for: Evaluating a 30-Hour Training Program for Community Health Workers on 4Ms Implementation in FQHCs Using the Kirkpatrick Model
Source: Healthcare (Basel). 2025 Oct 23;13(21):2677. doi: 10.3390/healthcare13212677 (PMC12608867; doi:10.3390/healthcare13212677)
Supplement: Supplementary file 1 [file healthcare-13-02677-s001.zip › healthcare-3910288-supplementary.pdf]

Supplementary Materials S1- 4Ms Intake Form

1. **Concerning What Matters** - Independence and Autonomy
   - What one thing do you feel interferes most with your day-to-day activities?
   - Do you need help with medications, bathing, toileting, dressing?
   - What brings you joy and satisfaction?
   - Have you thought about your preferences for end-of-life care?
   - What are your thoughts on advanced care planning?
   - Who supports you during a difficult time?
   - What challenges have you faced recently, and how can we support you with them?
     - Would you like more information

2. **Concerning Medications -** include over the counter medicines and Nutraceutical supplements as well as prescriptions
   - Do you understand the purpose of your medications?
   - Do you know how to take them? Are you experiencing any unwanted side effects?
   - What are your thoughts on medications and treatments for your conditions?
   - Would you like more education about your medication?
     - Would you like more information?

3. **Concerning Mentation**
   - How are you sleeping at night?
   - Are you having any trouble with your memory?
   - Are you able to focus, follow conversations and read the newspaper?
   - Do you feel depressed?
   - Are you the caretaker of someone with dementia?
     - Would you like more information?

4. **Concerning Mobility**
   - How far are you able to walk without stopping?
   - Do you use mobility aids such as a cane, walker, or wheelchair?
   - What are your mobility goals? Are you satisfied with your current level of mobility?-
     - Would you like more information? Web based Resources

Thank you for taking the time to let us know how we can support your healthcare goals!

Supplementary Materials S2- Final Exam for the Training Program

FINAL EXAM

NAME ____

INSTRUCTIONS: There are 20 questions, each worth 5 points. You have an hour to finish. Good luck!

1. Which of the following is NOT one of the 11 Core Competencies of CHWs?
   a. ____ Communication skills
   b. ____ Advocacy
   c. ____ Ability to speak another language
   d. ____ Outreach
   e. ____ Evaluation & Research

2. The primary role of the CHW in chronic disease management is to give the patients the medication that has been prescribed by the primary care physician.
   a. ____ True
   b. ____ False

3. Provide two resources for patients with dementia that are not offered in your Federally-Qualified Health Center. (*Hint: these resources can be from a community-based organization.)*
   1. ____
   2. ____

4. It is a requirement that a CHW must wear a headset in order to correctly perform Active Listening with a patient.
   a. ____ True
   b. ____ False

5. Which of the following are roles of working CHWs?
   a. ____ Health education
   b. ____ Health screenings
   c. ____ Health Risk Assessments
   d. ____ Transport of their own patients to clinic appointments
   e. ____ Medication management

6. Motivational Interviewing involves which of the following skills?
   a. ____ Open-ended questions
   b. ____ Affirmations to the patient
   c. ____ Speaking in English only
   c. ____ Re-phrasing what the patient says
   d. ____ Summarizing what the patient says

Supplementary Materials S3.1: Program Evaluation Survey

PROGRAM EVALUATION

1. First and last name________________________________

2. The content presented was appropriate for the intended audience
   I. Poor II. Fair III. Good IV. Very Good V. Excellent
3. The information presented could be applied to own practice
   I. Poor II. Fair III. Good IV. Very Good V. Excellent

4. The information is helpful in achieving professional goals
   I. Poor II. Fair III. Good IV. Very Good V. Excellent

PROGRAM IMPACT

5. Did the program meet your training expectations?
   I. Definitely did II. Somewhat did III. Not at all

6. Did the information presented today increase your knowledge on the subject matter?
   I. Definitely did II. Somewhat did III. Not at all

7. What new knowledge, information, and/or skills did you gain from participating in this activity?

8. Comments / Suggestions:

INSTRUCTOR

9. The speaker had clear knowledge in content area
   I. Poor II. Fair III. Good IV. Very Good V. Excellent

10. The speaker presented content that was consistent with objectives
    I. Poor II. Fair III. Good IV. Very Good V. Excellent

11. The speaker communicated effectively and was well prepared
    I. Poor II. Fair III. Good IV. Very Good V. Excellent

Supplementary Materials S3.2 Knowledge Survey

Feedback Form(Webinars, Professional Development)

Your participation in this survey is entirely voluntary, and your responses will be kept strictly confidential. The information you provide will be used solely for the purposes of program evaluation and will not be shared with anyone outside of the evaluation team. To ensure your privacy:

•All data will be anonymized and aggregated, so individual responses cannot be traced back to you.
•Personal identifiers will be removed before data analysis.
•Survey responses will be stored securely and accessed only by authorized personnel.
•Results will be reported in a way that does not identify any individual respondent.

By proceeding with this survey, you consent to the collection and use of your information as described. Thank you for your participation and valuable input.

*** Required**

1. Your Full Name*
2. Today's Date *
3. Date of Presentation *
4. Presentation Title *
5. Presenter *
6. Length of Presentation (in hours) *
7. Your Profession *

   • Faculty • Fellow • Resident • Student • CHW • Social Worker • Nurse • Medical Assistant • Other

8. Year in Program *
9. Select Your Employment Location *
   • Academic Institution • Critical Access Hospital • Other Clinical Training Site
   • Area Health Education Center • FQHC or Look-Alike • Rural Health Clinic • N/A • None of the Above

10. Your Degree/Certificate Program *

    • BS • BSN • Certificate-CHW • Certificate-CNA • Certificate-CNM • Certificate-CNS • Certificate-ENT • Certificate-interprofessional • Certificate-Other • Certificate-Pharmacy Aid • DNP • MA • MD • MS • MSN • NP • PhD • DDS • Not Applicable

11. Your Discipline*

    • Behavioral Health • Dentistry–General Dentistry • Medicine–Family Medicine • Public Health • Not Applicable • Other

12. Birth Year *

Supplementary Materials S3.3 Focus Group Interview Guide

CHW Focus Group Guide

Hello and welcome! Thank you all for taking the time to participate in this focus group today. We're here to gain insight into your experiences as community health workers, particularly focusing on the training you have received, your perceptions of that training, and any additional support you feel would be valuable for your work.

This discussion aims to understand better how we can improve training and support for community health workers like you, so your feedback is essential. This session is designed to be an open and safe space, and there are no right or wrong answers—your honest thoughts and experiences are what we're looking for. We encourage you to share freely and respect each other's viewpoints.

This focus group will take approximately one hour and will be recorded to ensure we accurately capture all your input. The information gathered will be kept confidential, and your names or personal details will not be associated with any responses in any reports or publications.

Background

1. What is your job title?
2. What are your current responsibilities?
3. How long have you been working as a Community Health Worker?
4. What inspired you to become a Community Health Worker?
5. Can you describe a typical day in your role?

The following questions will ask about the AHEC CHW Training you attended last month

6. Can you please describe your experience with the training?
7. How do you plan to apply the information learned in the training to your daily work?
8. How do you intend to refresh and stay updated on the topics covered in the training?
9. What additional trainings are you interested in receiving throughout the year to enhance your skills?
10. What other trainings did you participate in before the AHEC CHW Training?
11. Are there any topics you wish had been covered during the AHEC CHW Training that were not included?

Support

12. What types of support do you need to help you effectively incorporate what you learned in the training?
13. How are you currently implementing the knowledge and skills gained from the training in your work?
14. What challenges have you faced in applying the training to your job?
15. How do you feel about receiving refresher sessions on the training material?
    a. How often would you find refresher sessions beneficial?
16. How do you feel about receiving refreshers on the training?
    a. How often?
